# CD4^+^ Recent Thymic Emigrants Are Recruited into Granulomas during *Leishmania donovani* Infection but Have Limited Capacity for Cytokine Production

**DOI:** 10.1371/journal.pone.0163604

**Published:** 2016-09-22

**Authors:** John W. J. Moore, Lynette Beattie, Mohamed Osman, Benjamin M. J. Owens, Najmeeyah Brown, Jane E. Dalton, Asher Maroof, Paul M. Kaye

**Affiliations:** Centre for Immunology & Infection, Department of Biology and Hull York Medical School, University of York, York, United Kingdom; INRS—Institut Armand Frappier, CANADA

## Abstract

Recent thymic emigrants (RTEs) represent a source of antigen-naïve T cells that enter the periphery throughout life. However, whether RTEs contribute to the control of chronic parasitic infection and how their potential might be harnessed by therapeutic intervention is currently unclear. Here, we show that CD4^+^ recent thymic emigrants emerging into the periphery of mice with ongoing *Leishmania donovani* infection undergo partial activation and are recruited to sites of granulomatous inflammation. However, CD4^+^ RTEs displayed severely restricted differentiation either into IFNγ^+^ or IFNγ^+^TNFα^+^ effectors, or into IL-10-producing regulatory T cells. Effector cell differentiation in the chronically infected host was not promoted by adoptive transfer of activated dendritic cells or by allowing extended periods of post-thymic differentiation in the periphery. Nevertheless, CD4^+^ RTEs from infected mice retained the capacity to transfer protection into lymphopenic RAG2^-/-^ mice. Taken together, our data indicate that RTEs emerging into a chronically inflamed environment are not recruited into the effector pool, but retain the capacity for subsequent differentiation into host protective T cells when placed in a disease-free environment.

## Introduction

Visceral leishmaniasis (VL) is a systemic disease caused by the intracellular parasites *Leishmania donovani* and *L*. *infantum*, and can be fatal if left untreated. Experimental VL (EVL) in C57BL/6 mice is typified by organ-specific immune responses that result in a self-limiting disease in the liver and a chronic persisting infection with extensive immunopathology in the spleen. Associated with chronic infection of the spleen is a dramatic remodelling of stromal cells, including fibroblastic reticular cells (FRCs) follicular dendritic cells (FDCs), and of the marginal zone, collectively resulting in disruption to the spatial organisation of the splenic lymphoid tissue architecture [1]. As a consequence, the normal migration of lymphocytes and DCs into the periarteriolar lymphoid sheath (PALS) is impaired [1,2]. It has been proposed that this loss of structural organisation might lead to an inability to prime T cells during an on-going infection, contributing to more rapid clonal exhaustion of the initial cohort of antigen-responsive T cells. Although not formally reported for CD4^+^ T cells, exhaustion of CD8^+^ T cells during chronic *L*. *donovani* infection, in both mouse and humans has been observed [3,4].

In contrast to the spleen, the liver is a site of effective parasite clearance. Rapid uptake of parasites by liver-resident Kupffer cells (KCs) initiates the formation of granulomatous inflammation, a process characterized by the focal aggregation of predominantly mononuclear cells [5,6]. Within the granuloma, focused delivery of effector cytokines promotes the killing of intracellular parasites via a NO-dependent pathway, and subsequent to parasite clearance granulomas involute and normal liver architecture is essentially restored [6,7].

T cells play a major role in orchestrating an effective granulomatous response against *L*. *donovani* infection. T cell-deficient mice have unrestrained parasite growth and delayed and minimal hepatic inflammatory responses [8]. Cytokines important in resistance to hepatic infection are multiple and whilst dominated by those associated with Th1 responses, including IFNγ [9,10], TNFα [11,12], IL-12 [13–15], these also include cytokines associated with Th2 responses, such as IL-4 [16], IL-13 [17] and IL-1 [18]. Not surprisingly, enhancement of T cell responses e.g. by manipulation of co-stimulatory pathways such as CD40-CD40L [19] and OX40-OX40L [20] and CD80/86-CTLA4 [19–21] result in enhanced granuloma formation and increased parasite killing. In contrast, excess production of the regulatory cytokine IL-10 has been linked to the failure of the local T cell response to adequately control infection or to dampen the curative response. IL-10 production by CD4^+^ T cells that also co-express IFNγ [22–24] and by NK cells [25], are associated with increased parasite burden. In contrast, the frequency of CD25^+^ natural Tregs alters little during the course of experimental *L*. *donovani* infection [22]. Similarly, natural regulatory T cells were not detected within the spleens of human VL patients, whereas CD25^-^ CD4^+^ T cells accumulate IL-10 mRNA at this site [26,27].

Recent thymic emigrants (RTEs) represent a unique subset of peripheral T cells that have recently undergone thymic selection, and are defined by their period within the periphery and the expression of a variety of markers of activation. Studies using RAG2p-GFP mice, demonstrate that RTEs differ both phenotypically and functionally from mature peripheral T cells, and undergo a period of post-thymic maturation critical to acquiring functional capacity [28]. CD4^+^ RTEs displayed proliferative defects and reduced IL-2 secretion compared to mature T cells, whilst CD8^+^ RTEs displayed normal proliferation but an impaired capacity to secrete IFNγ [28]. Within immune replete animals, RTEs appear to be at a physiological disadvantage for entering the peripheral T cell niche, whereas their ability to do so is enhanced under conditions of lymphopenia [29]. IL-7 signalling may tune RTEs for limited proliferation but higher longevity [30]. Murine CD8^+^ RTEs generate lower frequencies of cytokine producing and long-lived memory cells compared to mature CD8^+^ T cells [31]. However, following a maturation period of ~2–3 weeks, rechallenge resulted in comparable effector responses between CD8^+^ RTE-derived memory cells and mature CD8^+^ memory cells, highlighting the important role that RTEs can play in maintaining both T cell homeostasis and immunity [31]. Although CD8^+^ RTE-derived memory cells have reduced granzyme B expression, their capacity to exert effector function appears to be compensated for by enhanced proliferative capacity [32]. RTEs have also been implicated in antiviral immunity, with CD8^+^ RTEs contributing to the virus-specific CD8^+^ T cell pool during chronic polyoma virus infection [33], whilst CD4^+^ RTEs contribute to the CD4^+^ memory T cell pool during influenza virus infection, even after virus clearance [34]. Most recently, CD8^+^ RTEs responding to low affinity altered peptide ligands have been shown to home to sites of bacteria-induced inflammation, again restricted in their ability to secrete effector cytokines [35] and it has been suggested that inflammation can overcome the tolerising effects of self antigen recognition [36]. Other studies have suggested that CD4^+^ RTEs are preferential precursors of regulatory T cells, suggesting a role for CD4^+^ RTEs in immune regulation [37] and perhaps in the persistence of chronic infections.

Here, we have addressed the question of whether CD4^+^ RTEs contribute to effector responses during established *L*. *donovani* infection. Using a microchimeric approach [38] that allows tracking of RTEs that emerge during on-going infection, we show that CD4^+^ RTEs display altered phenotype consistent with partial activation and are able to home to hepatic granulomas, yet fail to generate robust effector Th1-associated cytokine responses, even after adoptive transfer of activated DCs. These RTEs nevertheless express host protective capacity and effector function when transferred into lymphopenic RAG2^-/-^ mice, indicating that they retain *Leishmania*-antigen specificity and the potential for effector cell differentiation.

## Results

### CD4^+^ RTEs acquire an activated phenotype in the periphery of infected mice

**Fig 1:**
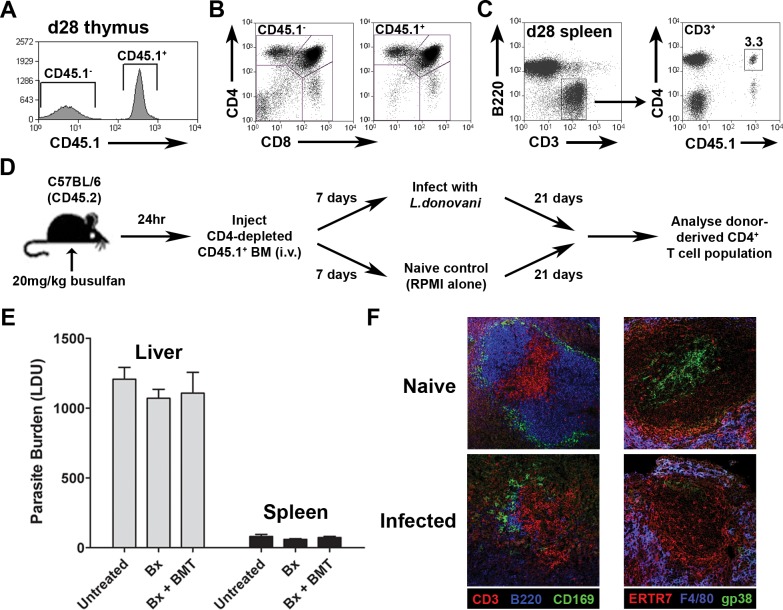
Busulfan chimeras as a valid approach to studying CD4^+^ RTE responses. CD4-depleted B6.CD45.1 BM cells were transferred into busulfan-treated CD45.2-expressing mice 24 hours after busulfan administration. Four weeks after BMT, thymi were removed and stained for CD45.1. Gates represent populations of CD45.1^+^ and CD45.1^-^ cells in the thymi of busulfan-treated animals (**A**). Thymi were also stained for CD4 and CD8. Gates represent the populations of double negative (DN; CD4^-^ CD8^-^), double positive (DP; CD4^+^ CD8^+^), CD4 single positive (CD4 SP; CD4^+^ CD8^-^) and CD8 SP (CD4^-^ CD8^+^) thymocytes within both CD45.1^-^ and CD45.1^+^ populations (**B**). Spleens were removed from busulfan chimeras 28 days after the BMT and stained for CD3, CD4 and CD45.1. CD3^+^ cells were gated upon (**C**—left panel) and the frequency of CD45.1^+^ CD4^+^ cells were measured (**C**—right panel). Schematic describes the experimental protocol used to study CD4^+^ RTEs in busulfan chimeras during experimental VL (**D**). Mice were treated with busulfan in the presence or absence of a CD4-depleted BMT. Untreated controls received saline solution instead of busulfan. Mice were infected with LV9 amastigotes 8 days post busulfan administration. Hepatic and splenic impression smears were collected at d29 post busulfan-treatment (d21 p.i.). Parasites were counted per 1000 cell nuclei and LDUs were calculated. Graph represents mean +/- SEM for 3 animals per group (**E**). Mice were treated with busulfan followed by a CD4-depleted BMT. Mice were either infected with LV9 amastigotes on day 7 post BMT or left uninfected. Spleens were removed from infected and naive mice at d28 post BMT (d21 p.i.). Frozen tissue sections were collected and stained for CD3 (T/NKT cells), B220 (B cells), CD169 (marginal metallophilic macrophages), ERTR7 (marginal zone macrophages), F4/80 (red pulp macrophages) and podoplanin /gp38 (fibroblastic reticular cells) (**F**).

In order to study the functional differentiation of RTEs that emerge into the periphery during chronic infection, we used the myeloablative drug busulfan [38] to create bone marrow micro-chimeras in which genetically identifiable RTEs emerge after the onset of disease-associated immunopathology (**Fig 1**). CD4-depleted BM cells from B6.CD45.1 mice (**S1 Fig**) were transferred into B6.CD45.2 recipients that had been treated with busulfan 24h previously (*B6*.*CD45*.*1 → B6*.*CD45*.*2*). Four weeks after BMT, analysis of thymi from these mice demonstrated efficient engraftment (**Fig 1A and 1B** and **S2 Fig**) and at this time donor-derived CD3^+^ CD4^+^ T cells (as well as CD3^+^CD8^+^ T cells) were also readily observed in the periphery (**Fig 1C**), along with donor-derived B cells and myeloid cells (**S3 Fig**). Kinetic analysis indicated that donor derived CD4^+^ T cells were not detectable in the periphery at day 14 post BMT, but were readily seen at day 28, consistent with data in the literature on the time taken for T cell development [39]. Herein, we operationally define this population of recently emergent donor-derived CD4^+^ T cells as CD4^+^ RTEs. Based on this observation and the kinetics of *L*. *donovani*-induced immunopathology [1,2,40], microchimeric mice were infected with *L*. *donovani* at day 7 post BMT, and analyzed at day 28 post BMT (day 21 post infection; p.i.; **Fig 1D**). Importantly, microchimeric mice responded to infection in an identical manner to untreated mice, as judged by liver and spleen parasite load (**Fig 1E**) and by immunohistochemical staining for splenic marginal zone and stromal cell integrity (**Fig 1F;** [1,2,40]).

**Fig 2:**
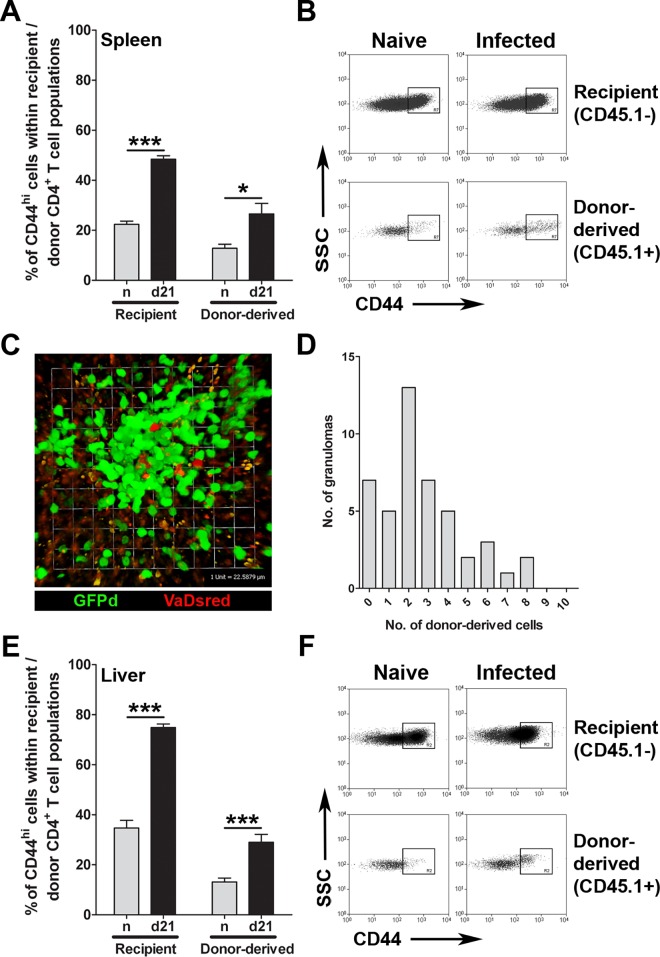
CD4^+^ RTEs are activated during EVL and RTEs are recruited to hepatic granulomas. CD45.2-expressing mice were treated with busulfan followed by a CD4-depleted CD45.1^+^ BMT 24 hours later. On day 7 post BMT mice were infected with LV9 amastigotes or left uninfected. Spleens were taken on day 28 post BMT (d21 p.i.) and stained for CD3, CD4, CD45.1 and CD44. Graph represents the percentage of CD44^hi^ cells in either the recipient CD4^+^ T cell population or the donor-derived CD4^+^ T cell population. Graph represents mean +/- SEM for 20 animals per group from 4 independent experiments (**A**). Flow plots represent gates used to determine CD44^hi^ CD4^+^ T cells (**B**). hCD2.GFPd mice were treated with busulfan followed by a CD4- and CD8-depleted VaDsRed BMT. Mice were infected with LV9 amastigotes on day 7 post BMT. Hepatic granulomas were imaged on d28 post BMT (d21 p.i.) from infected liver explants using 2-photon laser scanning microscopy (2P-LSM). Representative image displays a hepatic granuloma containing recipient T cells (green) and donor-derived RTEs (red) (**C**). Graph represents the distribution of donor-derived RTEs recruited to 3D z-stack reconstructions of 45 randomly selected hepatic granulomas (**D**). Busulfan chimeras were established as described above. Graph represents the percentage of CD44^hi^ cells in either the recipient CD4^+^ T cell population or the donor-derived CD4^+^ T cell population. Graph represents mean +/- SEM for 20 animals per group from 4 independent experiments (**E**). Flow plots represent gates used to determine CD44^hi^ CD4^+^ T cells (**F**).

We first compared the activation status of recipient CD4^+^ T cells with the CD4^+^ RTE population of donor origin, as judged by acquisition of a CD44^hi^ phenotype (**Fig 2**). In spleens, the frequency of CD44^hi^ recipient CD4^+^ T cells and CD44^hi^ CD4^+^ RTEs was increased during *L*. *donovani* infection, compared to that seen in naïve mice (p<0.0001 and p<0.03 respectively; **Fig 2A and 2B**). In livers, to assess whether RTEs could be recruited into *L*. *donovani*-induced granulomas, we made VaDsRed *→* GFPD microchimeras, wherein RTEs express the DsRed fluorochrome [41] and all recipient T cells express GFP [42]. RTEs were readily observable within hepatic granulomas by 2-photon imaging (**Fig 2C and 2D**). As this microscopic approach did not allow us to directly discriminate between CD4^+^ and CD8^+^ RTEs or activation status, we isolated hepatic mononuclear cells for analysis by flow cytometry, and demonstrated that intrahepatic CD4^+^ RTE cells (of which ~50% reside within granulomas; **S4 Fig**) also contained an increased frequency of CD44^hi^ cells (p<0.0001; **Fig 2E and 2F**). Collectively, these data show that CD4^+^ RTEs become activated in the periphery of mice infected with *L*. *donovani* and can accumulate within granulomas.

### CD4^+^ RTEs fail to undergo functional differentiation

**Fig 3:**
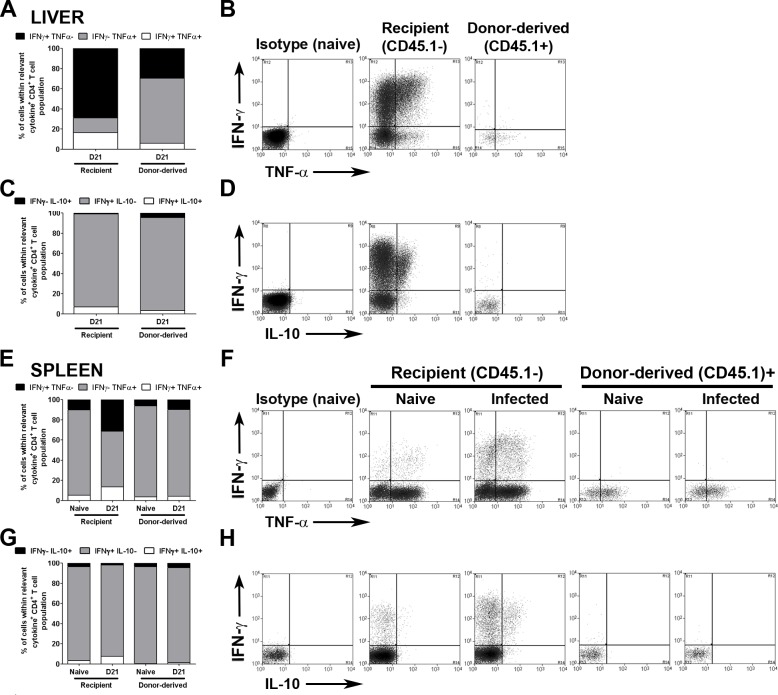
CD4^+^ RTEs display impaired Th1 cytokine responses during EVL. Busulfan chimeras were established as described in Fig 2. Spleens and livers were taken on day 28 post BMT (d21 p.i.), stimulated with PMA and ionomycin, and stained for CD3, CD4, CD45.1, IFNγ, TNFα and IL-10. Graphs represent the distribution of IFNγ and TNFα expression (**A&E**), or the distribution of IFNγ and IL-10 expression (**C&G**) from recipient CD4^+^ T cells or donor-derived CD4^+^ RTEs in both the liver (**A&C**) and the spleen (**E&G**). Distributions were calculated by converting the frequency of cytokine expression into a proportion of all cytokine expressing recipient/donor-derived CD4^+^ T cells, with regards to the relative cytokine axis. Graph represents the mean for 15 animals per group from 3 independent experiments. Flow plots represent gates used to determine IFNγ^+^, TNFα^+^ and IL-10^+^ expression by CD4^+^ T cells in both the liver (**B&D**) and the spleen (**F&H**).

We next compared recipient CD4^+^ T cells with donor-derived CD4^+^ RTEs (gating shown in **S5 Fig**) for their ability to express cytokines associated with effector and regulatory function. We used PMA / ionomycin to re-stimulate cytokine production ex vivo to avoid introducing further TCR-dependent signals and to capture the commitment of T cells for cytokine production that had been shaped in the periphery. In d21-infected livers, a proportion of recipient CD4^+^ T cells had differentiated sufficiently to have the capacity to produce IFNγ (28.6 ± 2.2% of total recipient CD4^+^ T cells), or both IFNγ and TNFα (7.7 ± 1.8% of total recipient CD4^+^ T cells). In relative terms, these populations accounted for 85.3 ± 1.9% (IFNγ^+^, 68.9 ± 2.0%; IFNγ^+^TNFα^+^, 16.3 ± 2.1%) of all cytokine-producing cells detectable under these restimulation conditions **(Fig 3A and 3B)**. In contrast, within the CD4^+^ RTE population, IFNγ^+^TNF^+^ cells were infrequent (<1%), and TNFα single producing cells contributed the bulk of cytokine producing CD4^+^ RTEs (64.4 ± 5.4%; **Fig 3A and 3B**). IFNγ^+^IL-10^+^ CD4^+^ T cells are also present in the liver, these cells representing Th1 cells that have received prolonged activation by conventional dendritic cells (cDCs) [43]. Of cytokine producing recipient CD4^+^ T cells, 6.9 ± 1.6% co-expressed IFNγ and IL-10 (3.0 ± 0.9% of total recipient CD4^+^ T cells), whereas amongst CD4^+^ RTEs that were capable of producing IFNγ, <0.5% had differentiated sufficiently to produce IL-10 (**Fig 3A and 3D**). Although for comparison we attempted to study cytokine production by RTEs in the livers of naïve mice, yields were insufficient for robust analysis.

In contrast to liver, the spleen is unable to clear its parasite load, a phenomenon associated with changes in lymphoid tissue microarchitecture [1,2,40] as well as the presence of immunoregulatory cytokines including IL-10 [22,23]. RTE’s were also sufficiently abundant in the spleen for a comparative cytokine analysis between these cells in naïve and infected mice. IFNγ^+^ (11.6 ± 0.6% of total recipient CD4^+^ T cells) and IFNγ^+^TNFα^+^ (4.8 ± 0.8% of total recipient CD4^+^ T cells) recipient CD4^+^ T cells were less abundant in infected spleens than infected livers, and accounted for only 44.8 ± 3.7% of all cytokine producing cells, with the remaining recipient CD4^+^ T cells making TNFα alone (**Fig 3E and 3F**). Importantly, the frequency of IFNγ^+^TNFα^+^ and IFNγ^+^ recipient CD4^+^ T cells increased significantly in response to *L*. *donovani* infection (p = 0.0008; p<0.0001 respectively). TNF single-producing cells were also the dominant population amongst cytokine producing CD4^+^ RTEs (naïve, 90.2 ± 1.7%; infected, 86.0 ± 2.8%; **Fig 3E and 3F**). In contrast to recipient CD4^+^ T cells, the frequency of IFNγ^+^TNFα^+^ and IFNγ^+^ CD4^+^ RTEs did not alter significantly in response to infection, both in terms of proportion of total CD4^+^ RTEs (IFNγ^+^TNFα^+^, p = 0.6; IFNγ^+^, p = 0.3, data not shown) and proportion of cytokine producing CD4^+^ RTEs (IFNγ^+^TNFα^+^, p = 0.7; IFNγ^+^ p = 0.4; **Fig 3E and 3F**). Th1 cells that had differentiated to co-produce IFNγ and IL-10 were restricted to the recipient population of splenic CD4^+^ T cells (1.0 ± 0.2% of total; 7.5 ± 1.6% of cytokine producers), and increased significantly in infected spleens compared to naïve mice (p<0.0001; **Fig 3G and 3H**). CD4^+^ RTEs displayed minimal co-expression of IFNγ and IL-10 in both naïve and infected spleens (**Fig 3G and 3H**). We conclude that CD4^+^ RTEs in spleen or liver poorly acquire the capacity for multifunctional effector cytokine responses in the periphery of *L*. *donovani-*infected mice.

We estimate that RTEs, as operational defined here, would have spent up to 14 days in the periphery before analysis. We therefore considered the possibility that the limited effector response displayed by CD4^+^ RTEs might reflect insufficient time for peripheral maturation, given that recent studies have suggested that up to 3 weeks is required for this process to fully occur [28,31]. To address this, we analyzed a further set of busulfan chimeras at d28 p.i. (day 35 post BMT), when the cohort of RTEs would have spent up to 21 days in the periphery. Despite this extended time period, a similar pattern of cytokine expression was observed within both the liver and the spleen (**S6 Fig**). CD4^+^ RTEs displayed restricted capacity for IFNγ expression compared to recipient CD4^+^ T cells, although the capacity to differentiate into IFNγ^+^IL-10^+^ cells was marginally improved. In the spleen, limited effector cell differentiation was again observed (p = 0.15 IFNγ^+^TNFα^-^; p = 0.2 IFNγ^+^IL-10^-^ vs. naive). Furthermore, cells displaying singular TNFα expression comprised the majority of cytokine-expressing CD4^+^ RTEs **(S6 Fig)**.

### CD4^+^ RTEs produce effector cytokines in response to strong TCR stimulation

**Fig 4:**
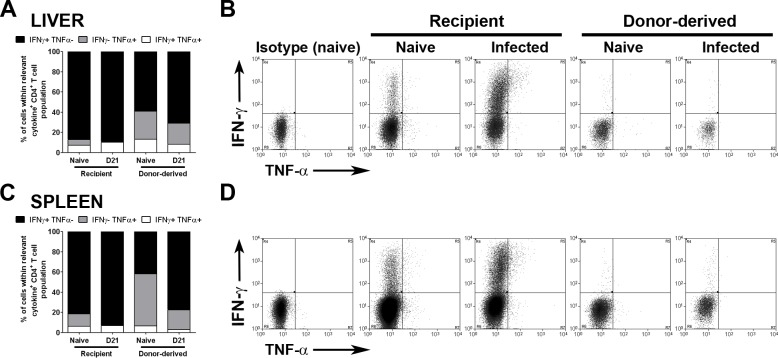
CD4^+^ RTEs are capable of responding to TCR stimulation. Busulfan chimeras were established as described in Fig 2. Spleens and livers were taken on day 28 post BMT (d21 p.i.) and CD4^+^ cells were collected by MACS. CD4^+^ cells were stimulated with anti-CD3 and anti-CD28 for 12 hours and stained for CD3, CD4, CD45.1, IFNγ and TNFα. Graphs represent the distribution of IFNγ and TNFα expression from recipient CD4^+^ T cells or donor-derived CD4^+^ RTEs in both the liver (**A**) and the spleen (**C**). Distributions were calculated by converting the frequency of cytokine expression into a proportion (x%) of all cytokine expressing recipient/donor-derived CD4^+^ T cells (100%), with regards to the relative cytokine axis. Graph represents the mean for 5 animals per group from 1 experiment. Flow plots represent gates used to determine IFNγ^+^ and TNFα^+^ expression by CD4^+^ T cells in both the liver (**B**) and the spleen (**D**).

To further investigate why CD4^+^ RTEs were unable to produce effector cytokines in chronically infected mice, we tested their ability to respond to direct TCR stimulation ex vivo. CD4^+^ T cells were purified from naïve and *L*. *donovani*-infected mice and stimulated with plate-bound anti-CD3 and anti-CD28. In both the liver and spleen, recipient CD4^+^ T cells exhibited an IFNγ-dominated response with the majority of cytokine-producing cells expressing IFNγ in the absence of TNFα expression **(Fig 4A–4D)**. The proportion of cells with this phenotype was significantly increased in CD4^+^ T cells isolated from *L*. *donovani* infected mice (p<0.0001 in both spleen and liver; **Fig 4A–4D**), as was the frequency of IFNγ^+^TNFα^+^ co-producers (spleen, p = 0.0005; liver, p<0.0001; **Fig 4A–4D**). CD4^+^ RTEs from naïve mice activated with anti-CD3/CD28 were not as heavily biased towards IFNγ expression, compared to the recipient CD4^+^ T cell population **(Fig 4A–4D**), but IFNγ expression was clearly more evident than for the same cells stimulated ex vivo with PMA / ionomycin (c.f. **Fig 3A and 3E**) and the frequency of IFNγ^+^TNFα^-^ CD4^+^ RTEs in the spleen increased significantly in *L*. *donovani* infected mice (p = 0.0005; **Fig 4A–4D**). The frequency of IFNγ^+^TNFα^-^ CD4^+^ RTEs in the liver of infected mice also increased but this change was not of a similar extent. Co-production of IFNγ and TNFα by CD4^+^ RTEs was less affected by choice of stimulus, however (**Fig 4A–4D** vs. **Fig 3A and 3E**). These data indicate that RTEs isolated at d28 post BMT and having spent ~14 days in the periphery of either naïve or infected mice have matured sufficiently to produce IFNγ^+^ in response to strong TCR / costimulation in vitro, but that that far fewer RTEs acquire this capacity in vivo in *L*. *donovani* infected mice.

### Adoptive DC therapy fails to promote RTE differentiation

**Fig 5:**
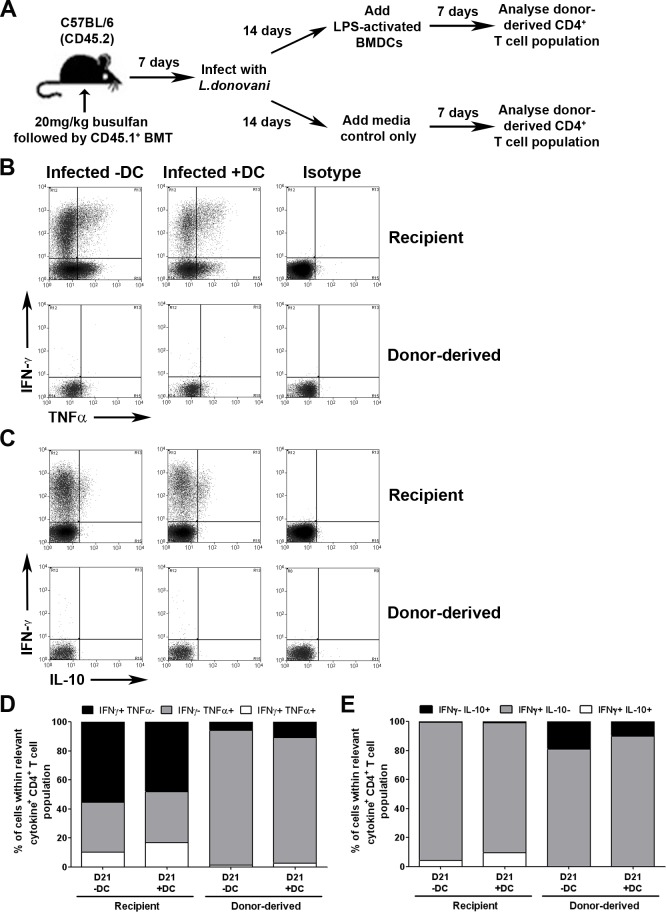
Administration of LPS activated BMDCs does not improve CD4^+^ RTE cytokine responses. Busulfan chimeras were established as described in Fig 2. 1x10^6^ LPS-activated BMDCs were transferred into busulfan chimeras on d21 post BMT (d14 p.i.). Spleens were taken on day 28 post BMT (d21 p.i.), stimulated with PMA and ionomycin, and stained for CD3, CD4, CD45.1, IFNγ, TNFα and IL-10 (**A**). Flow plots represent gates used to determine IFNγ^+^ and TNFα^+^ expression (**B**), or IFNγ^+^ and IL-10^+^ expression (**C**) by CD4^+^ T cells in the spleen. Graphs represent the distribution of IFNγ and TNFα expression (**D**), or the distribution of IFNγ and IL-10 expression (**E**) from recipient CD4^+^ T cells or donor-derived CD4^+^ RTEs in the spleen. Distributions were calculated by converting the frequency of cytokine expression into a proportion (x%) of all cytokine expressing recipient/donor-derived CD4^+^ T cells (100%), with regards to the relative cytokine axis. Graph represents the mean for 5 animals per group from 1 experiment.

Competition for antigen presentation by DCs and / or DC-derived cytokines may underpin the inability of naïve T cells to gain access to the signals that promote full differentiation [44–46]. To address the possibility that a lack of sufficient stimulation was responsible for the lack of RTE differentiation into cells with the capacity to produce IFNγ in *L*. *donovani-*infected mice, we adoptively transferred LPS-activated bone marrow-derived DCs into *B6*.*CD45*.*1 → B6*.*CD45*.*2* microchimeric mice at d14 p.i and examined the T cell response 7 days later (**Fig 5A**). In mice infected with *L*. *donovani*, transfer of LPS-activated DCs significantly enhanced the frequency of recipient CD4^+^ T cells producing both IFNγ^+^TNFα^+^ (10.0 ± 1.5% vs. 16.7 ± 1.8%; p<0.02) and IFNγ^+^IL-10^+^ (4.3 ± 0.5% vs. 9.5 ± 1.3%; p<0.02), demonstrating the functionality of the transferred DCs **(Fig 5B–5E)**. However, transfer of DCs had no significant impact on the ability of CD4^+^ RTEs to differentiate into effectors with the capacity to produce cytokines other than TNFα (**Fig 5D–5E**). These data suggest that competition for DC-derived signals is unlikely to underpin the failure of RTEs to acquire a capacity for IFNγ production during infection.

### CD4^+^ RTEs can provide host protective immunity in a lymphopenic host

**Fig 6:**
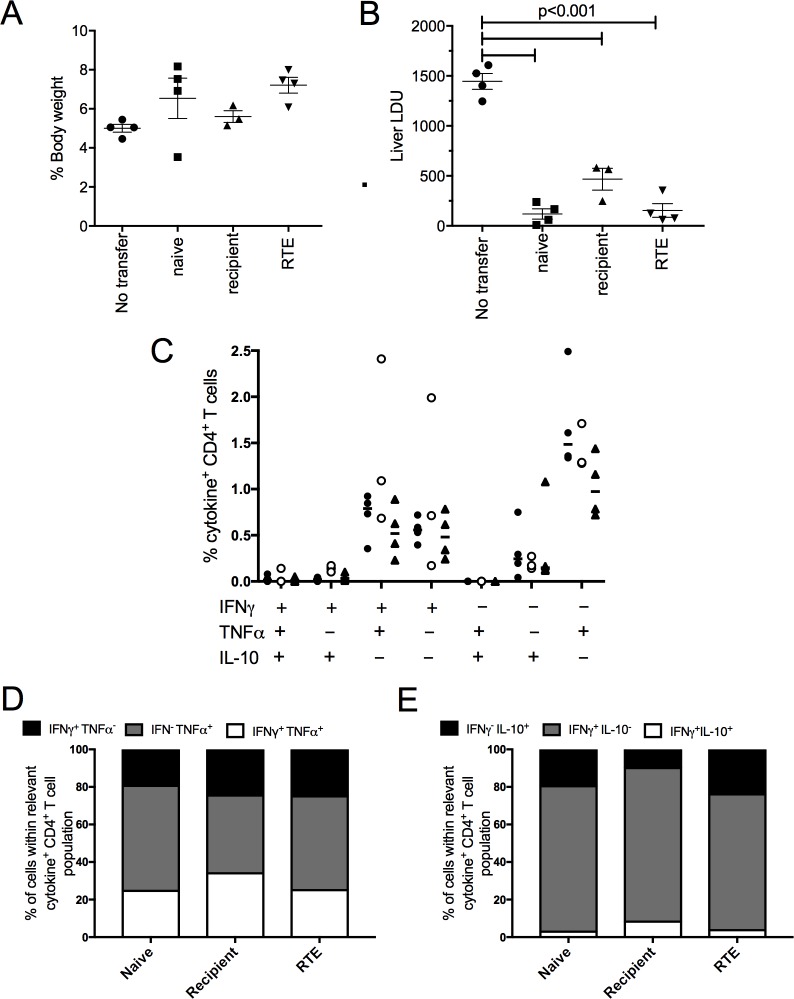
CD4^+^ RTE adoptively protect RAG2^-/-^ mice against *L*. *donovani* infection. RAG2-/- recipients were adoptively transferred with CD4^+^ T cells obtained from naïve B6 mice (naive) or recipient CD4^+^ T cells or CD4^+^ RTEs obtained from d28-infected chimeric mice. All recipients were infected with *L*. *donovani* at day 1 post transfer and at day 28 post infection, mice were assessed for hepatomegaly (**A**) and parasite load (**B**). Data were analyzed by ANOVA with using Tukey post test, and are shown as liver weight as % body weight (A) and as LDUs (B) for 3–4 animals per group from 1 experiment. CD4^+^ T cells recovered from the spleens of infected RAG mice at day 28 post infection were assayed for intracellular IFNγ, TNFα and IL-10 production after PMA / ionomycin stimulation. Data are shown as the frequency of single, double and triple-cytokine producing cells within the total CD4^+^ T cell population for naïve CD4^+^ T cells (closed circles), recipient CD4^+^ T cells (open circles) and CD4^+^ RTEs (closed triangles) (n = 3–4) (**C**) and as the distribution of IFNγ and TNFα producing cells (**D**) and IFNγ and IL-10 producing cells (**E**), calculated as in Fig 3.

Our collective data suggest that whereas the commitment to enhanced effector cytokine production is a feature of the polyclonal CD4^+^ T cell population in infected mice, this does not extend to the CD4^+^ RTE population. To rule out the possibility that intra-thymic presentation of *Leishmania* antigens had led to clonal deletion of *Leishmania*-reactive T cells from the RTE repertoire and to confirm whether these RTE could differentiate into effector cells in the environment of a naïve lymphopenic host, we sorted recipient CD4^+^ T cells and CD4^+^ RTEs from infected mice and CD4^+^ T cells from naïve mice and adoptively transferred each population into naïve RAG2^-/-^ recipients (**Fig 6**). One day post transfer, RAG2^-/-^ mice were infected with *L*. *donovani* and the outcome of infection assessed at day 28 p.i., as a measure of functional antigen-specific T cell responses. Adoptive transfer of naïve CD4^+^ T cells, recipient CD4^+^ T cells from infected mice and CD4^+^ RTE’s all reconstituted protection in these RAG recipients, with no differences in the extent of hepatomegaly. Of note, the frequency of FoxP3^+^ CD4^+^ T cells within the adoptively transferred host at day 28 post infection was not indicative of the level of adoptive protection provided (CD4^+^ naïve, 3.5±0.87; CD4^+^ “recipient”, 0.2±0.07; CD4^+^ RTE, 1.47±0.56) or different between mice receiving CD4^+^ RTEs and mature recipient CD4^+^ T cells. To confirm the cytokine-producing capacity of RTEs that mediate protection in infected RAG2^-/-^ mice, we stained splenic T cells isolated from these mice for IFNγ, TNFα and IL-10. We found no significant difference in cytokine production (quantitatively or qualitatively) between transferred naïve CD4^+^ T cells, recipient CD4^+^ T cells from infected mice or CD4^+^ RTEs from infected mice, indicating that under these conditions RTEs have the capacity to acquire effector function to the same extent as other CD4^+^ T cell populations (**Fig 6C–6E**). Together these data confirm that CD4^+^ RTEs retain antigen specificity for *L*. *donovani*, provide good protection against infection and undergo efficient effector cell differentiation when transferred and allowed to expand in a lymphopenic environment. Of note the proportions of IFNγ^+^ and IFNγ^+^TNFα^+^ cells in these RAG recipients was similar to that seen for recipient cells in busulfan chimeras (comparing **Fig 6D** and **Fig 3E**) suggesting that both these single and dual cytokine-producing cell populations may be associated with protective function.

## Discussion

Immune responses during the chronic stages of *L*. *donovani* infection are compromised by the collective actions of regulatory cytokines including IL-10 and TGFβ, by changes to the tissue microenvironment, and by T cell intrinsic functional defects such as anergy and / or exhaustion (reviewed in [7]). Although RTEs already in the periphery may contribute to the primary immune response to infection, the fate of recent thymic emigrants that emerge from the thymus into this complex environment has not been previously studied, but has important implications for the design of therapeutic strategies to enhance host protective immunity during chronic infection. Here report that CD4^+^ RTE’s become partially activated and home to sites of infection in the liver, yet fail to acquire full effector function in the environment of the chronically infected host. Nevertheless, these cells have retained functional host protective capacity as demonstrated by transfer into lymphopenic hosts, suggesting that their effector function could be mobilized for protective benefit.

Previous studies have suggested that disruption to the spatial organisation of the spleen during chronic EVL might result in an inability to initiate priming of naïve CD4^+^ T cells. Our data shows the frequency of CD4^+^ RTEs expressing the activation marker CD44 increases significantly in *L*. *donovani*-infected animals, suggesting that CD4^+^ RTEs are at least partially activated during an ongoing infection. Within the population of cytokine producing RTEs, however, differentiation into multifunctional cytokine-expressing CD4^+^ T cells is impaired. Such multi-functional T cells have been implicated as the major effector cell population in studies aimed at identifying correlates of vaccine-induced protection in leishmaniasis [47], though whether such cells are more potent than single IFNγ -producing cells in the context of natural resistance in the intact host is yet to be formally determined. Although ~30% of RTEs had the capacity to express TNFα, arguing against a deficiency in CD4^+^ RTEs to produce any cytokines, the frequency of TNFα^+^ CD4^+^ RTEs did not alter in response to infection and cytokine production was generally of lower level that that seen in recipient CD4^+^ T cells from the same mice. This suggests that TNFα expression by CD4^+^ RTEs is not a response to *L*. *donovani* infection per se but rather reflects the intermediate state of “licencing” for TNFα production shown by RTEs relative to their single positive thymic precursors and mature naïve CD4^+^ T cells [48]. Mechanistically, this is likely due to differences between these cells in chromatin remodelling at cytokine loci [49].

RTEs have been shown to require a period of post-thymic maturation of ~14 days before they become fully functional T cells [28]. Prior to this maturation process, CD4^+^ RTEs display a reduced proliferative capacity and poor IL-2 expression compared to mature naïve T cells. This state of reduced responsiveness appears to resemble the response of CD4^+^ RTEs in *L*. *donovani*-infected mice. Thus, the lack of effector CD4^+^ RTEs may reflect a lack of post-thymic maturation during chronic *L*. *donovani* infection. Access to SLOs was shown to be essential for post-thymic maturation [29] and CD4^+^ RTEs were clearly detectable within *L*. *donovani*-infected spleens. However, it is possible that the disrupted splenic architecture observed during chronic infection may hamper the ability of CD4^+^ RTEs to undergo post thymic maturation in *L*. *donovani*-infected spleens. Although dramatic splenomegaly accompanies *L*. *donovani* infection, this is not associated with lymphopenia [50]. Thus, as described by Fink and colleagues [28,29,31], RTEs are likely to remain at a competitive disadvantage relative to mature peripheral T cells, whereas on non-competitive transfer into a lymphopenic host, RTEs appear to be able to fully provide host protection and undergo effector cell differentiation. It is difficult experimentally to dissect whether this reflects the increased capacity to RTEs to differentiate in the absence of competition from other T cells and /or the lack of the potentially immunosuppressive environment associated with chronic infection. However, our attempts to overcome competition in immunocompetent infected mice by adoptive transfer of DC suggest that functional maturation associated with expansion in a lymphopenic host may be a dominant factor, and suggest that interventions that mimic this effect in the infected host may provide a means to enhance host protection mediated by RTEs.

The activation but poor cytokine production of CD4^+^ RTEs during infection also displays some hallmarks of bystander activation. During chronic *L*. *donovani* infection, CD4^+^ T cells have been shown to undergo bystander activation and proliferation [50], and intravital imaging studies have demonstrated that Bacille Calmette-Guérin (BCG)- and *L*. *donovani* -induced hepatic granulomas respectively recruit CD4^+^ and CD8^+^ T cells independently of antigen specificity [41,51]. We show here that *L*. *donovani* granulomas also recruit RTEs. Homing to the sites of inflammation, mediated by increased VLA-4 expression, has been described as a property of CD8^+^ RTEs and preferential homing may allow RTEs to exert a greater effector function than predicted by their level of cytokine expression [35].

Others have reported that CD4^+^ RTEs may be biased towards a regulatory function [37]. Although we found that the frequency of FoxP3 expressing cells was slightly but not significantly higher in our RTE population compared to non-RTE cells derived from infected mice (“recipient” CD4^+^ T cells), it was still lower than that seen in the naïve splenic CD4^+^ T cell pool, indicating no preferential expansion of CD4^+^FoxP3^+^ RTEs had occurred. Indeed, RTEs made very little IL-10 under the stimulation conditions we used in these studies. These data reinforce earlier studies that indicate that in both mouse and humans, much of the regulatory capacity of the CD4^+^ T cell subset during VL lies within Th1 cells that have been induced to further differentiation into IFNγ^+^ IL-10^+^ cells [22,24,27,43]. Collectively, our data argue against the notion that RTEs emerging during chronic *L*. *donovani* infection preferentially differentiate into regulatory cells.

From the perspective of an emerging RTE, *Leishmania* antigens presented in infected target tissues such as spleen and liver might be perceived as self tissue-specific antigens. In this regard, a recent study demonstrated that compared to mature cells, OT-I and OT-II RTEs recognising OVA in RIP-mOVA transgenic mice were defective in both proliferation and cytokine production, expressed markers of anergy, and were susceptible to Treg induced suppression. However, these “tolerance prone” RTEs were fully competent to become effector cells when recognising OVA in the context of alum-induced inflammation [36]. Contrasting this with our data, it would appear that either the highly inflammatory environment generated by *L*. *donovani* infection, which involves a mixture of both Th1, Th2 and regulatory cytokines, is unable to convert RTEs into effector cells (i.e. the wrong type of inflammation), or the impact of such tolerance-breaking cytokines is negated by the gross microenvironmental changes described above (i.e. the right inflammation but in the wrong environment). Although both mechanisms would limit effector function in the infected immunocompetent host, the ability of these RTEs to subsequently provide full, rather than diminished, protection when transferred into RAG hosts indicates that functional potential within the RTE population is not indelibly curtailed.

In summary, the data presented shows that during an on-going *L*. *donovani* infection, CD4^+^ RTEs can become activated but are unable to differentiate into mature IFNγ-producing effectors cells. CD4^+^ RTE expression of activation markers downstream of TCR signalling would suggest that CD4^+^ RTEs are encountering APCs during infection but are not becoming efficiently primed. This may be due to tolerance or due to an inability of RTEs to undergo appropriate post thymic maturation in *L*. *donovani*-infected spleens. This inefficient priming of naive CD4^+^ RTEs, in combination with local exhaustion [3] and increased apoptosis of T cells [52], suggests that there is minimal replenishment of the T cell pool during on-going infection, thus limiting effector T cell responses. Our data also suggest that the outcome of infection might rest on the fate of T cells primed early in infection, prior to the onset of overt pathology and immune regulation. This conclusion is in keeping with studies of CD4^+^ T cell responses following *L*. *major* [53] and *Salmonella* [54] infection, that collectively suggest that continued re-exposure of pre-existing memory and / or effector CD4^+^ T cells to persistent antigen is important for the maintenance of effector function. Furthermore, whilst immunotherapeutic options exist that might allow such T cells to overcome immune regulation [7,55], interventions promoting the subsequent recruitment of RTEs into the effector response may also have therapeutic benefit.

## Methods

### Mice and Infections

6–8 week old Female C57BL6 (B6) mice (Charles River, Margate, UK), congenic B6.CD45.1 (CD45.1) mice, VaDsRed mice, which were originally a kind gift from Dimitris Kioussis (NIMR, Mill Hill, UK), and hCD2 GFPd mice (GFPd: NIMR, Mill Hill, UK) were used. *Leishmania donovani* (LV9) parasites were maintained in B6.RAG1^-/-^ mice. 3x10^7^ amastigotes were injected intravenously (i.v.) to initiate infection. Experiments were approved by the University of York Animal Welfare and Ethics Review Body and performed under UK Home Office license (PPL 60/4377; Immunology and Immunopathology of leishmaniasis). Mice were killed by cervical dislocation prior to tissue collection, as described below.

### Busulfan chimeras

Busulfan (Laboratories Pierre Fabre, France) was administered i.p. to mice at a concentration of 20mg/kg. CD4^+^ cells (and CD8^+^ cells where stated) were removed from bone marrow (BM) cells using CD4 (and CD8) microbeads and a MACS^®^ separation column according to manufacturer’s instructions (Miltenyi Biotec, Germany). 5x10^6^ CD4-depleted BM cells suspended in RPMI 1640 media were injected i.v. into busulfan-treated mice 24 hours after busulfan administration.

### Adoptive transfers

B6. RAG2^-/-^ recipients were injected i.p. with 1x10^6^ flow sorted mature CD4^+^ T cells or CD4^+^ RTEs (isolated from d28-infected busulfan chimeras) or with CD4^+^ T cells isolated from naïve mice. 1 day post transfer, these mice were infected with *L*. *donovani* and tissue parasite burden was assessed 28 days later.

### Flow cytometry

Livers were perfused with ice-cold PBS via the hepatic portal vein, and placed into ice-cold RPMI 1640 media. Liver tissue was passed through a 100μm cell strainer, washed twice (423g, 7 min), and hepatic mononuclear cells isolated on a 33% Percoll gradient (centrifuged at 693g, 12mins, slow acceleration/deceleration). Spleens were excised and placed directly in ice-cold RPMI 1640 media. Spleen tissue was passed through a 100μm cell strainer and washed twice (311g, 5 min). Red blood cells were removed from hepatic mononuclear and splenic cells by rapid treatment with Gey’s solution for 1 min before thorough washing. Hepatic mononuclear or splenic cells were plated into 96-well plates (~1 x 10^6^ cells/well) and incubated in FACS buffer (PBS supplemented with 1% FCS and 5mM EDTA) containing anti-CD16/32 Ab for 10 min on ice. After washing, cells were stained in 100μl FACS buffer containing fluorochrome-labelled antibodies specific for CD3e (500A2), CD4 (RM4-5), CD8 (53–6.7), B220 (RA3-6B2), CD11c (N418), CD11b (M1/70), Gr-1 (RB6-8C5), CD44 (IM7), or CD45.1 (A20), or appropriate isotype controls (antibodies from eBioscience or BD Biosciences). Labelled cells were acquired on a Cyan flow cytometer using Summit software (Beckman Coulter).

### Generation of BMDCs

10^7^ BM cells were incubated in complete RPMI 1640 media (RPMI 1640 media supplemented with containing 10% GM-CSF, 10% FCS, 2mM L-glutamine, 100U/ml penicillin and 100μg/ml streptomycin) at 37°C for 8–9 days. Media was checked regularly throughout and was replaced at least once during the 8–9 day incubation period with fresh complete RPMI-1640 media. For transfer of LPS-activated BMDCs into mice, 1μg/ml of LPS was added to BMDCs on day 8 for the final 24hr of culture. BMDCs were removed from the culture, washed and counted. LPS-activated BMDCs were resuspended in plain RPMI 1640 media and 1x10^6^ cells per mouse were injected i.v. For antigenic stimulation, on day 8, BMDCs were incubated with freeze/thawed *L*. *donovani* amastigotes at a ratio of 1:100 for 24 hr. On day 9, BMDCs were removed from the culture, washed and counted. BMDCs were plated into a 24-well plate at ~4x10^6^ cells/well. 1μg/ml of LPS was added to BMDCs for a further 6hr before BMDCs were washed thoroughly with complete RMPI-1640 media.

### Intracellular cytokine analysis

Hepatic mononuclear or splenic cells were plated into a 24-well plate (5 x 10^6^ cells/well) in RPMI 1640 media supplemented with 10% FCS. Cells were incubated with 50ng/ml of PMA and 500ng/ml of ionomycin at 37°C for 3 hr. 10μg/ml of Brefeldin A was added for a further 4 hr, before extracellular antibody labelling, fixation in 2% PFA (10 min on ice), permeabilization with 0.5% saponin and staining for IFNγ (XMG1.2), IL-10 (JES5-16E3) and TNFα (MP6-XT22). For antigenic stimulation, hepatic mononuclear or splenic cells (responder cells) were added to BMDCs at a ratio of ~2:1. Responder cells and BMDCs were cultured together for 3h at 37°C. 10μg/ml of Breeding A was added for a further 4 hr. before extracellular antibody labelling, fixation in 2% PFA (10 min on ice), permeabilization with 0.5% saponin, and staining for IFNγ, IL-10 and TNFα. For direct TCR stimulation, 48-well flat-bottom plates were coated overnight at RT with 10 μg/ml of anti-CD3e and 10 μg/ml anti-CD28 in PBS in a volume of 100 μl per well. The next day plates were washed with PBS and 6×10^5^ MACS-purified CD4^+^ T cells were seeded per well in 200 μl of RPMI 1640 supplemented with 10% FCS. CD4^+^ T cells were MACS-purified from hepatic mononuclear cells or splenic cells using CD4 microbeads and a MACS^®^ separation column according to manufacturer’s instructions (Miltenyi Biotec, Germany). Hepatic or splenic CD4^+^ T cells were cultured with the plate-bound anti-CD3e and anti-CD28 for 7h at 37°C. 10μg/ml of Brefeldin A was added for a further 5h before extracellular antibody labelling, fixation in 2% PFA (10 min on ice), permeabilization with 0.5% saponin and staining for IFNγ, IL-10 and TNFα. Labelled cells were analysed on a Cyan flow cytometer with Summit software (Beckman Coulter).

### 3D-imaging of hepatic explants

Freshly removed liver tissue was placed in 35mm coverslip-bottom Petri dishes (MatTek corporation), kept moist with ice cold RPMI 1640 and imaged on an inverted LSM 510 multiphoton microscope (Carl Zeiss Microimaging). Images were acquired with a 40x 1.1 water immersion objective and fluorescence excitation provided by a Chameleon XR Ti:sapphire laser (Coherent) tuned to ~870-900nm. For 3D analysis, 20–40μm Z stacks were acquired with a Z distance of 2–3μm. Images were rendered and analysed using Volocity software (Improvision).

### Quantification of parasite burden

Impression smears were collected for liver and spleen on superfrost glass slides. Slides were fixed with 100% methanol and air-dried. Fixed impression smears were labelled with Giemsa staining solution for 20–30 min. Parasite burdens were quantified using light microscopy and expressed as LDU values, calculated as the number of parasites per 1000 host cell nuclei x organ weight.

### Immunofluorescence of splenic architecture

Frozen spleen sections (~12 μm thick) were acetone fixed and stained with mAbs to MMM (MOMA-1; Acris Antibodies), MZM (ERTR9; Bachem), T cell zone FRC (Alexa Fluor 488–conjugated podoplanin; gp38; eBioscience), CD3 (500A2; eBioscience), B220 (RA3-6B2; eBioscience) and F4/80 (BM8; eBioscience), and MAdCAM-1 biotin conjugated (Serotec). Fluorochrome-conjugated goat anti-rat antibodies were used for detection of purified antibodies whilst fluorochrome-conjugated, streptavidin-conjugated antibodies were used for the detection of biotin-conjugated antibodies. Sections were counterstained with DAPI, mounted in Pro-long Gold anti-fade reagent (Invitrogen) and visualized using a Carl Zeiss inverted LSM META 510 confocal microscope. For distributional analysis of VaDsred^+^ RTEs within infected livers, liver tissue was fixed in 4% paraformaldehyde (PFA) for two hours before overnight incubation in 30% sucrose. Tissue was embedded in Optimal Cutting Temperature (OCT) medium (Sakura) and frozen on dry ice. Confocal microscopy was performed on 8–10 μm frozen sections. Sections were counterstained with DAPI, mounted in Pro-long Gold anti-fade reagent (Invitrogen) and visualized using a Carl Zeiss inverted LSM META 510 confocal microscope.

### Statistical Analyses

Sample sizes for animal experiments were calculated based on existing published or pilot data and were chosen to be able to detect changes in the primary parameter of 20% with 80% power and p<0.05. Animals were allocated to treatment groups and down stream processing of samples was conducted blind to treatment group. For normally distributed data, ANOVA with Tukey post test was applied, or students t test was applied for two sample comparisons.

## Supporting Information

S1 Fig Phenotype of donor T cells used to establish busulfan chimeras. B6.CD45.1 BM cells were harvested from naïve mice and labeled with CD4 microbeads. Labeled BM cells were passed through a MACS column according to manufacturer’s instructions (Miltenyi Biotec). Pre-sort and post-sort BM cells were stained for CD3 and CD4. Flow plots represent the gate used to determine the frequency of CD3^+^ CD4^+^ T cells within the BM. Graph represents the percentage of CD4^+^ T cells within pre-sort and post-sort BM. Graph represents mean +/- SEM from 7 independent experiments.
(TIF)

S2 Fig Phenotype of thymocytes in busulfan chimeras. CD4-depleted B6.CD45.1 BM cells were transferred into busulfan-treated CD45.2-expressing mice 24 hours after busulfan administration. On day 28 post BMT, thymi were removed and stained for CD45.1, CD4 and CD8. Graph represents the frequency of DN, DP, CD4 SP and CD8 SP thymocytes within both recipient CD45.1^-^ and donor-derived CD45.1^+^ populations. Graph represents mean +/- SEM for 10 animals per group, from two independent experiments *p<0.05.
(TIF)

S3 Fig Phenotype of donor-derived leucocytes post in busulfan chimeras. CD4-depleted B6.CD45.1 BM cells were transferred into busulfan-treated CD45.2-expressing animals 24 hours after busulfan administration. On day 28 post BMT, spleens were removed and stained for CD3, B220, Gr-1, CD11b and CD11c. Graph represents the frequency of CD3^+^, B220^+^, Gr-1^+^ CD11b^-^, Gr-1^+^ CD11b^+^ and CD11c^+^ CD11b^+^ cells within the donor-derived CD45.1^+^ population. Graph represents mean ± SEM for 3 animals per group.
(TIF)

S4 Fig Identification of donor derived T cells in hepatic granulomas in busulfan chimeras. hCD2.GFPd mice were treated with busulfan followed by a CD4- and CD8-depleted VaDsRed BMT. Mice were infected with LV9 amastigotes on day 7 post BMT and liver tissue taken on day 28 post BMT (d21 p.i.). Confocal microscopy was performed on PFA-fixed liver tissue sections that had been counterstained with DAPI. 100 VaDsred^+^ cells were analyzed and their position within or outside a granuloma was recorded. A granuloma was defined as an accumulation of 10 or more cell nuclei. Graph represents the distribution of 200 donor-derived RTEs within the livers of 4 mice, from 2 independent experiments.
(TIF)

S5 Fig Gating strategy to identify CD4^+^ T cells in busulfan chimeras. To study both recipient CD4^+^ T cells and donor-derived CD4^+^ RTEs within the liver, events were first gated by their pulse width profile. A FSC v SSC lymphocyte gate was then set and all CD3^hi^ CD4^+^ events were selected from this population in order to exclude CD3^lo^ NKT cells. Of the CD3^hi^ CD4^+^ events, CD45.1 expression was used to determine donor-derived and recipient populations. CD3^hi^ CD4^+^ CD45.1^-^ events were defined as recipient CD4^+^ T cells and CD3^hi^ CD4^+^ CD45.1^+^ events were defined as donor-derived CD4^+^ RTEs. A similar process was used within the spleen except the total CD3^+^ CD4^+^ population was gated on for further CD45.1-based separation.
(TIF)

S6 Fig CD4^+^ T cell cytokine production in extended duration busulfan chimeras. CD45.2-expressing mice were treated with busulfan followed by a CD4-depleted CD45.1^+^ BMT 24 hours later. On day 7 mice were infected with LV9 amastigotes or left uninfected. Spleens and livers were taken on day 35 post BMT (d28 p.i.), stimulated with PMA and ionomycin, and stained for CD3, CD4, CD45.1, IFNγ, TNFα and IL-10. Graphs represent the distribution of IFNγ and TNFα expression (**A&C**), or the distribution of IFNγ and IL-10 expression (**B&D**) from recipient CD4^+^ T cells or donor-derived CD4^+^ RTEs in the spleen. Distributions were calculated by converting the frequency of cytokine expression into a proportion (x%) of all cytokine expressing recipient/donor-derived CD4^+^ T cells (100%), with regards to the relative cytokine axis. Graph represents mean +/- SEM for 5 animals per group from 1 experiment.
(TIF)
