# Supplementary figures and images for: CD4+ Recent Thymic Emigrants Are Recruited into Granulomas during Leishmania donovani Infection but Have Limited Capacity for Cytokine Production
Source: PLoS One. 2016 Sep 22;11(9):e0163604. doi: 10.1371/journal.pone.0163604 (PMC5033337; doi:10.1371/journal.pone.0163604)

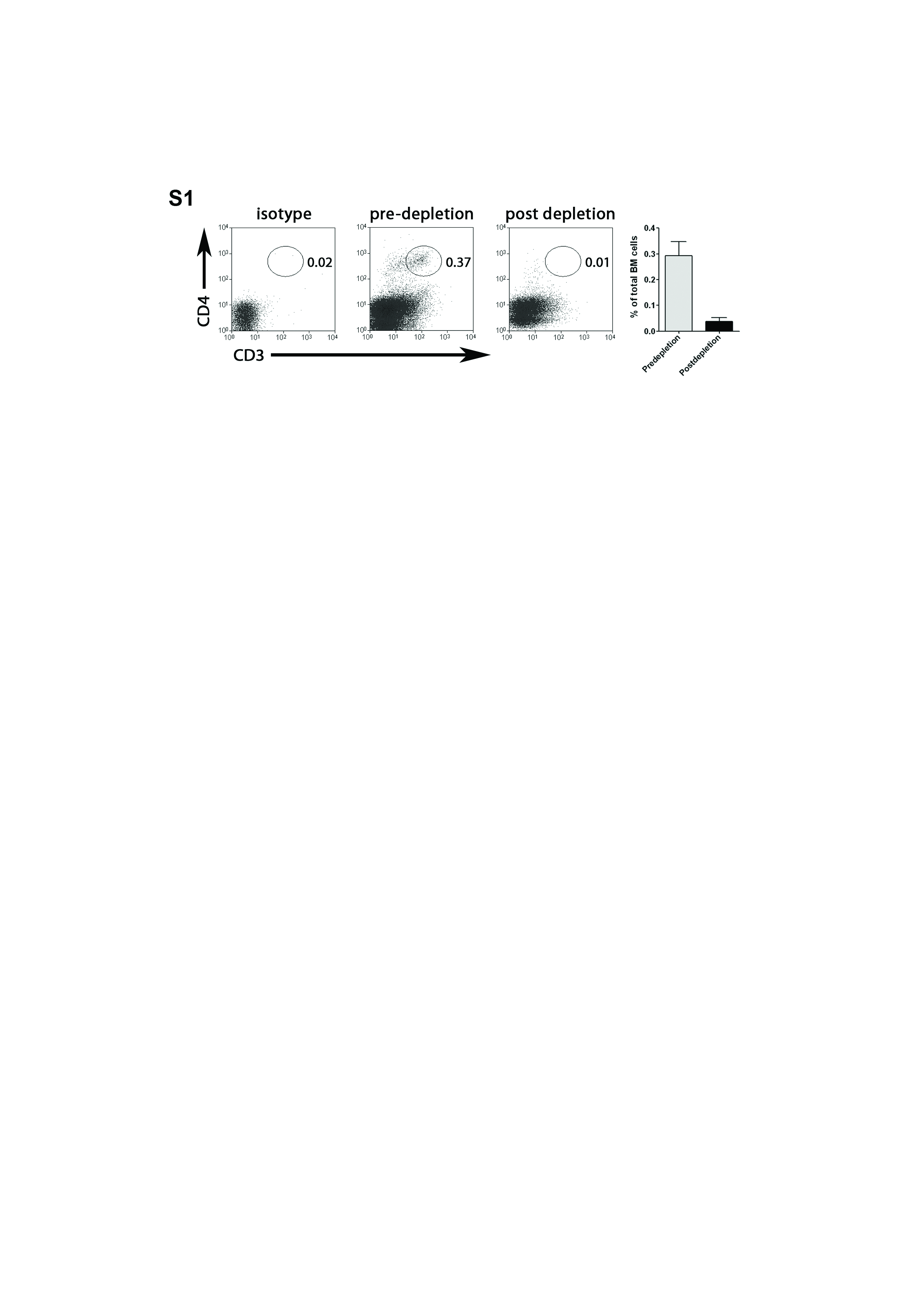

Supplement: S1 Fig — B6.CD45.1 BM cells were harvested from naïve mice and labeled with CD4 microbeads. Labeled BM cells were passed through a MACS column according to manufacturer’s instructions (Miltenyi Biotec). Pre-sort and post-sort BM cells were stained for CD3 and CD4. Flow plots represent the gate used to determine the frequency of CD3+ CD4+ T cells within the BM. Graph represents the percentage of CD4+ T cells within pre-sort and post-sort BM. Graph represents mean +/- SEM from 7 independent experiments. (TIF) [file pone.0163604.s001.tif]

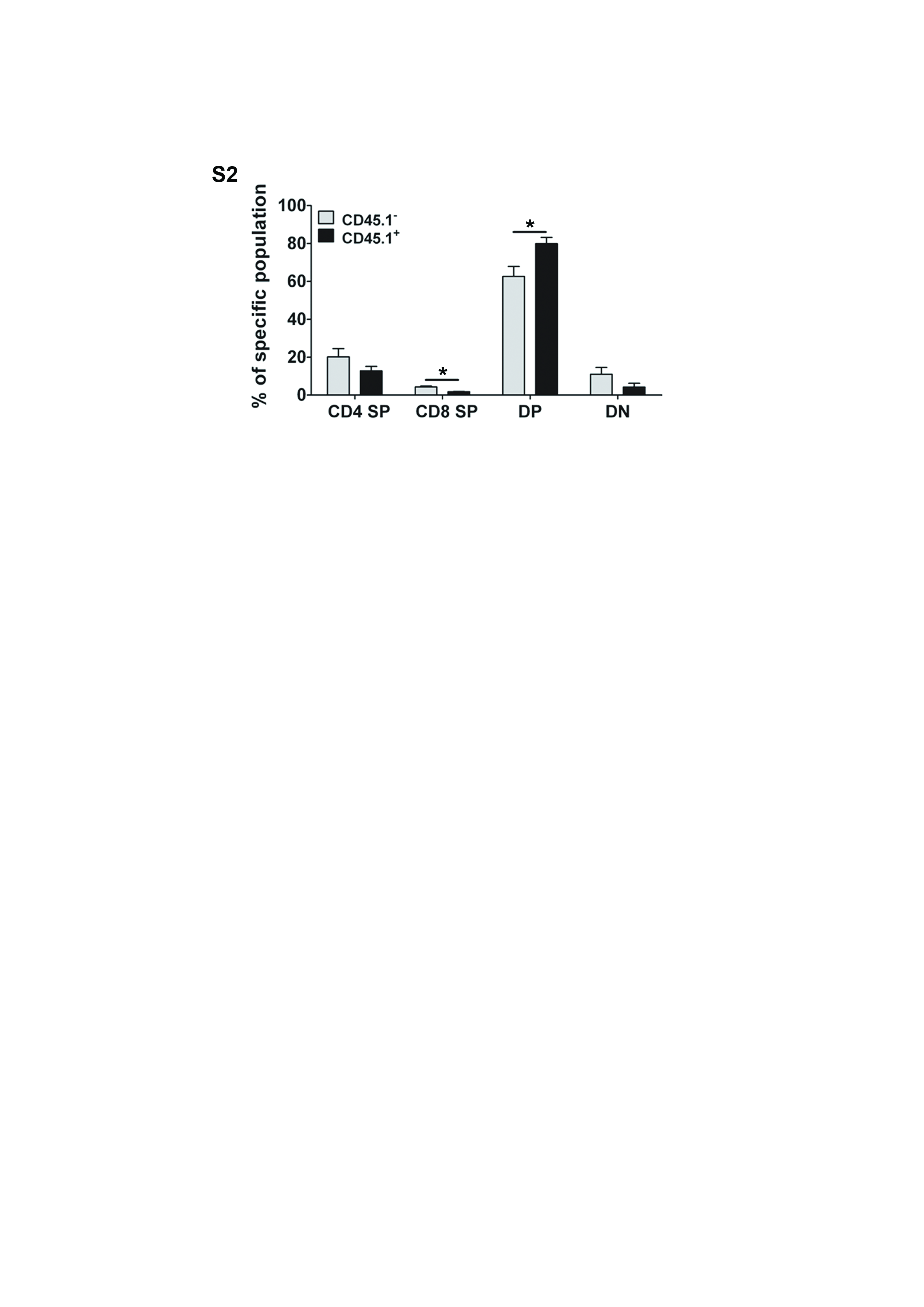

Supplement: S2 Fig — CD4-depleted B6.CD45.1 BM cells were transferred into busulfan-treated CD45.2-expressing mice 24 hours after busulfan administration. On day 28 post BMT, thymi were removed and stained for CD45.1, CD4 and CD8. Graph represents the frequency of DN, DP, CD4 SP and CD8 SP thymocytes within both recipient CD45.1- and donor-derived CD45.1+ populations. Graph represents mean +/- SEM for 10 animals per group, from two independent experiments *p<0.05. (TIF) [file pone.0163604.s002.tif]

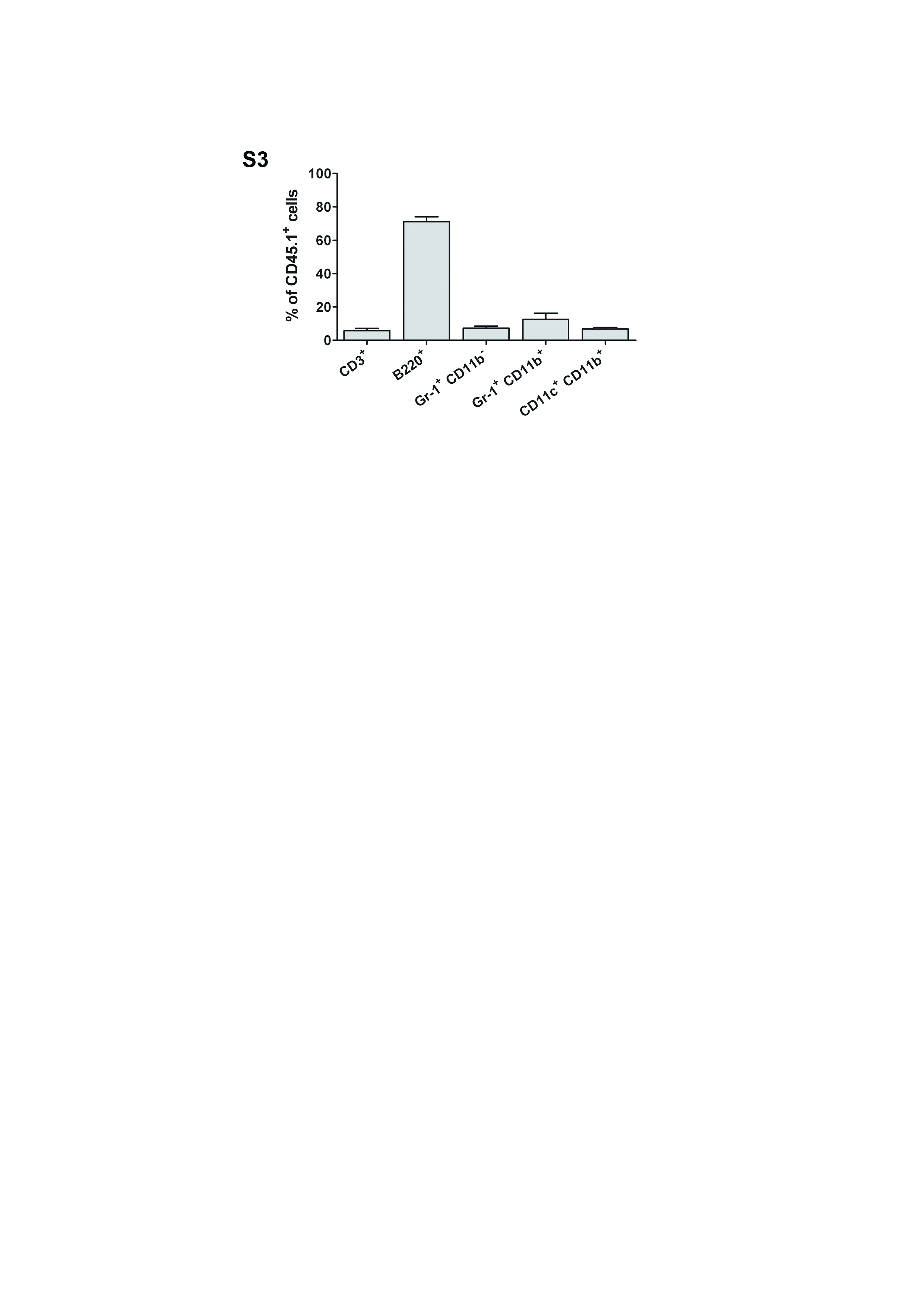

Supplement: S3 Fig — CD4-depleted B6.CD45.1 BM cells were transferred into busulfan-treated CD45.2-expressing animals 24 hours after busulfan administration. On day 28 post BMT, spleens were removed and stained for CD3, B220, Gr-1, CD11b and CD11c. Graph represents the frequency of CD3+, B220+, Gr-1+ CD11b-, Gr-1+ CD11b+ and CD11c+ CD11b+ cells within the donor-derived CD45.1+ population. Graph represents mean ± SEM for 3 animals per group. (TIF) [file pone.0163604.s003.tif]

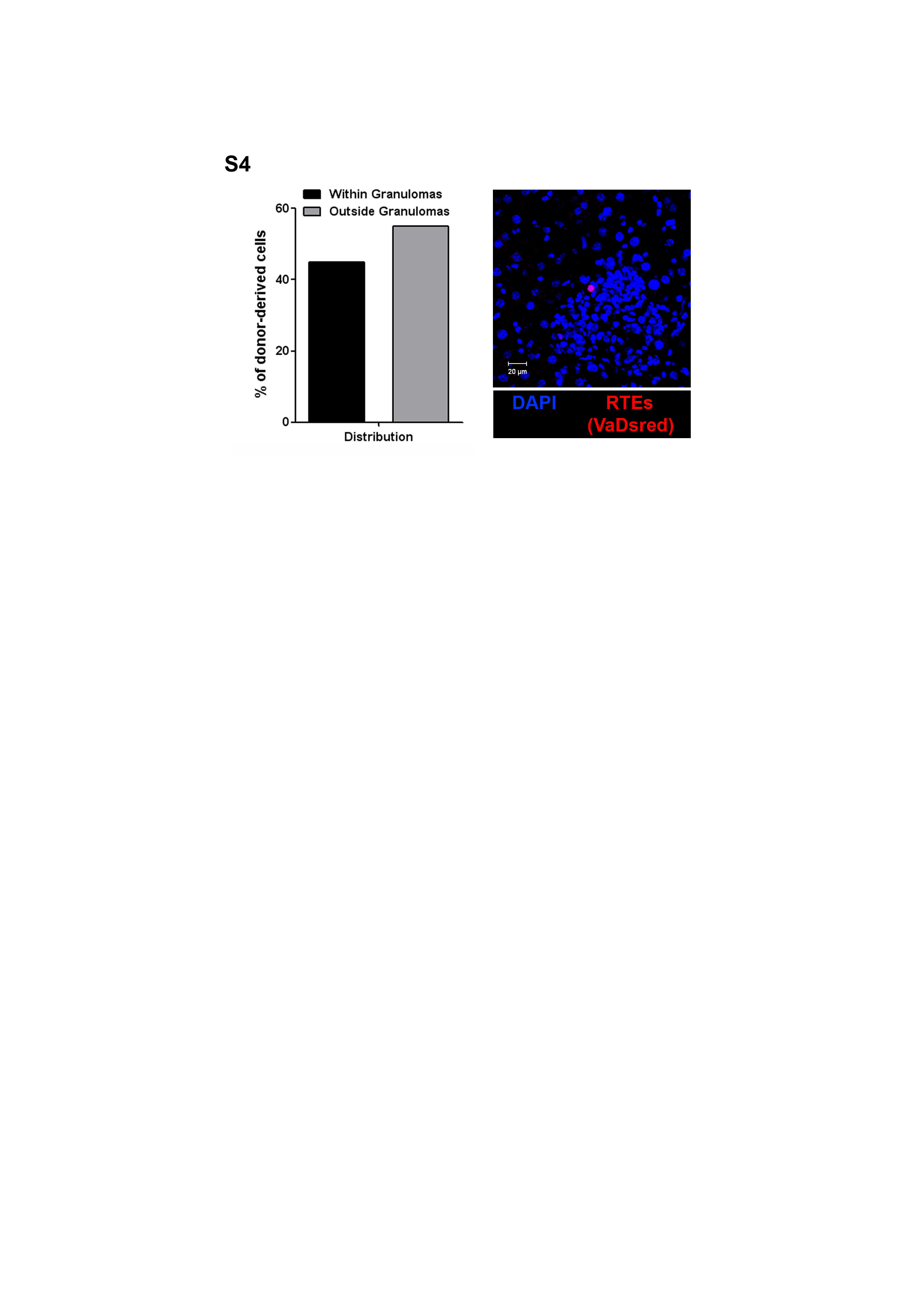

Supplement: S4 Fig — hCD2.GFPd mice were treated with busulfan followed by a CD4- and CD8-depleted VaDsRed BMT. Mice were infected with LV9 amastigotes on day 7 post BMT and liver tissue taken on day 28 post BMT (d21 p.i.). Confocal microscopy was performed on PFA-fixed liver tissue sections that had been counterstained with DAPI. 100 VaDsred+ cells were analyzed and their position within or outside a granuloma was recorded. A granuloma was defined as an accumulation of 10 or more cell nuclei. Graph represents the distribution of 200 donor-derived RTEs within the livers of 4 mice, from 2 independent experiments. (TIF) [file pone.0163604.s004.tif]

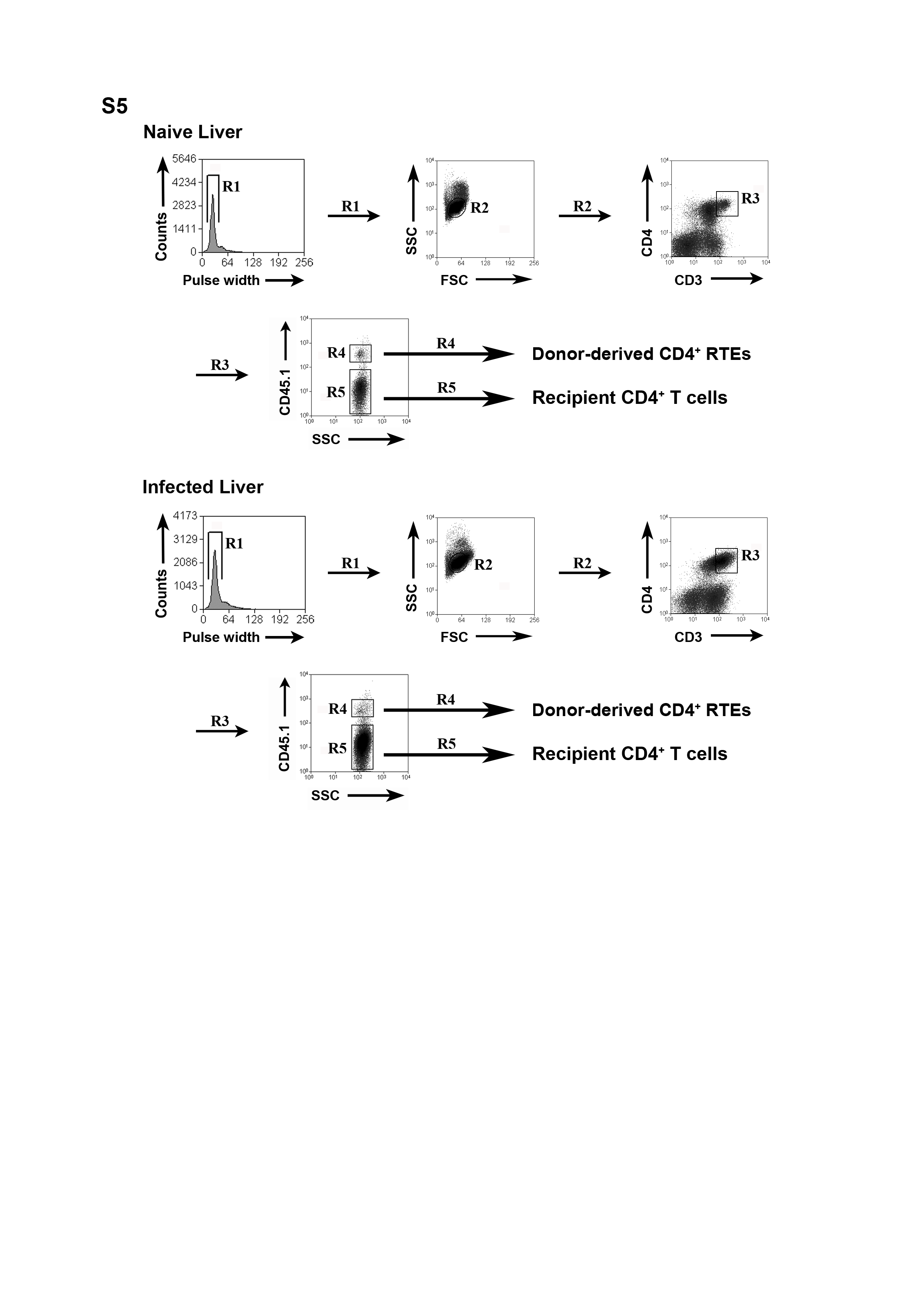

Supplement: S5 Fig — To study both recipient CD4+ T cells and donor-derived CD4+ RTEs within the liver, events were first gated by their pulse width profile. A FSC v SSC lymphocyte gate was then set and all CD3hi CD4+ events were selected from this population in order to exclude CD3lo NKT cells. Of the CD3hi CD4+ events, CD45.1 expression was used to determine donor-derived and recipient populations. CD3hi CD4+ CD45.1- events were defined as recipient CD4+ T cells and CD3hi CD4+ CD45.1+ events were defined as donor-derived CD4+ RTEs. A similar process was used within the spleen except the total CD3+ CD4+ population was gated on for further CD45.1-based separation. (TIF) [file pone.0163604.s005.tif]

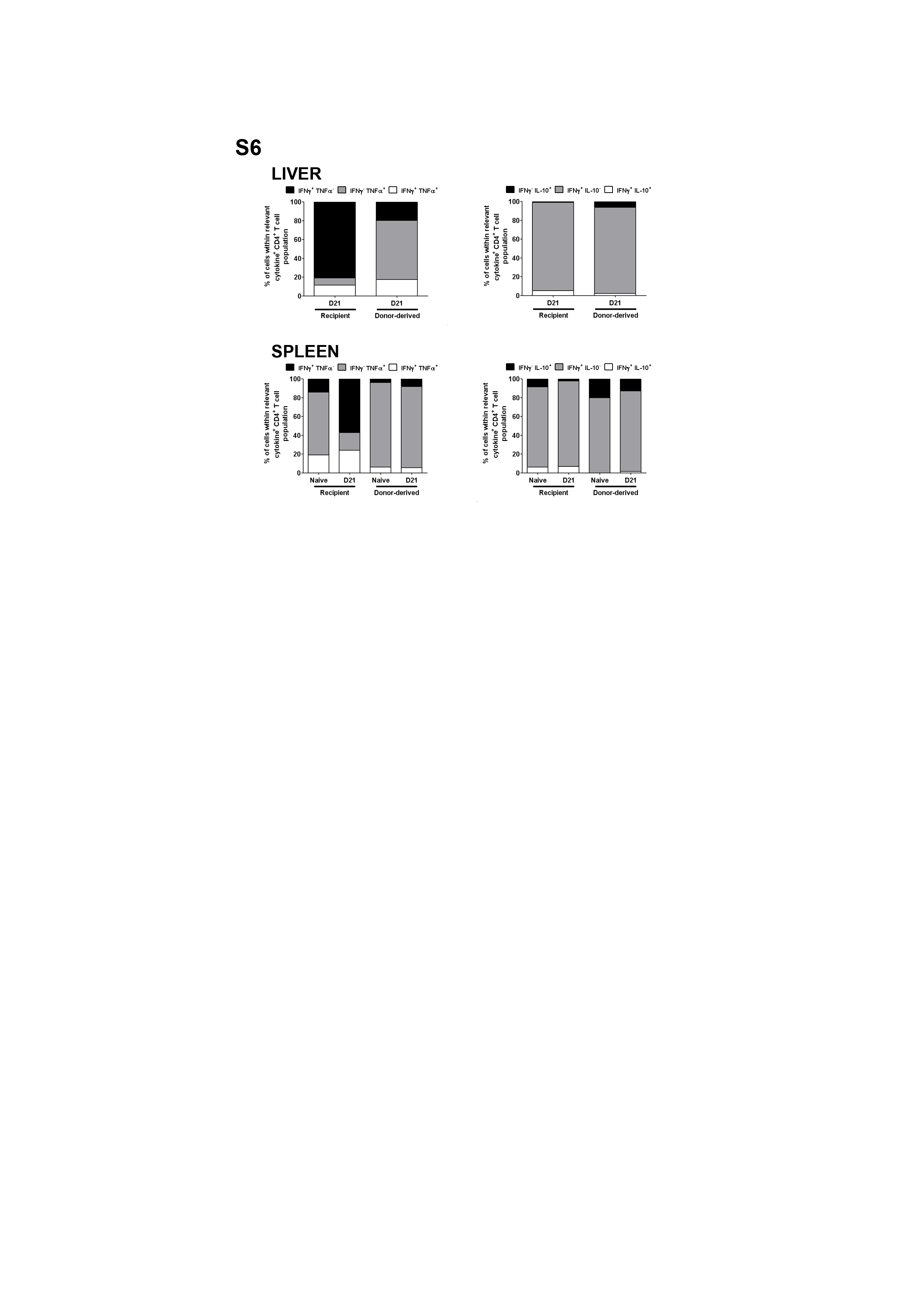

Supplement: S6 Fig — CD45.2-expressing mice were treated with busulfan followed by a CD4-depleted CD45.1+ BMT 24 hours later. On day 7 mice were infected with LV9 amastigotes or left uninfected. Spleens and livers were taken on day 35 post BMT (d28 p.i.), stimulated with PMA and ionomycin, and stained for CD3, CD4, CD45.1, IFNγ, TNFα and IL-10. Graphs represent the distribution of IFNγ and TNFα expression (A&C), or the distribution of IFNγ and IL-10 expression (B&D) from recipient CD4+ T cells or donor-derived CD4+ RTEs in the spleen. Distributions were calculated by converting the frequency of cytokine expression into a proportion (x%) of all cytokine expressing recipient/donor-derived CD4+ T cells (100%), with regards to the relative cytokine axis. Graph represents mean +/- SEM for 5 animals per group from 1 experiment. (TIF) [file pone.0163604.s006.tif]
